# Inhaled beta-2 agonist salbutamol and acute lung injury: an association with improvement in acute lung injury

**DOI:** 10.1186/cc3971

**Published:** 2006-01-11

**Authors:** Sanjay Manocha, Anthony C Gordon, Ebrahim Salehifar, Horacio Groshaus, Keith R Walley, James A Russell

**Affiliations:** 1 Clinical/Research Fellow, Critical Care Research Laboratories, Centre for Cardiovascular and Pulmonary Research, University of British Columbia, Vancouver, BC, Canada; 2 Clinical/Research Fellow, Critical Care Research Laboratories, Centre for Cardiovascular and Pulmonary Research, University of British Columbia, Vancouver, BC, Canada; 3 Pharmacist, Critical Care Research Laboratories, Centre for Cardiovascular and Pulmonary Research, University of British Columbia, Vancouver, BC, Canada; 4 Research Assistant, Critical Care Research Laboratories, Centre for Cardiovascular and Pulmonary Research, University of British Columbia, Vancouver, BC, Canada; 5 Professor of Medicine, Critical Care Research Laboratories, Centre for Cardiovascular and Pulmonary Research, University of British Columbia, Vancouver, BC, Canada; 6 Professor of Medicine, Critical Care Research Laboratories, Centre for Cardiovascular and Pulmonary Research, University of British Columbia, Vancouver, BC, Canada

## Abstract

**Introduction:**

β2 agonists have several properties that could be beneficial in acute lung injury (ALI). We therefore chose to study the effect of inhaled β2 agonist use (salbutamol) on duration and severity of ALI.

**Methods:**

We undertook a retrospective chart review of 86 consecutive mechanically ventilated patients with ALI, who had varying exposure to inhaled salbutamol. The cohort was divided into two groups according to the average daily dose of inhaled salbutamol they received ('high dose' ≥ 2.2 mg/day and 'low dose' <2.2 mg/day). Severity of ALI and non-pulmonary organ dysfunction was compared between the groups by calculating the days alive and free of ALI and other organ dysfunctions.

**Results:**

The high dose and low dose groups received a mean of 3.72 mg and 0.64 mg salbutamol per day, respectively. The high dose salbutamol group had significantly more days alive and free of ALI than the low dose group (12.2 ± 4.4 days versus 7.6 ± 1.9 days, *p *= 0.02). There were no associations between dose of β agonist and non-pulmonary organ dysfunctions. High dose salbutamol (*p *= 0.04), APACHE II score (*p *= 0.02), and cause of ALI (*p *= 0.02) were independent variables associated with number of days alive and free of ALI in a multivariate linear regression model.

**Conclusion:**

Our retrospective study suggests that salbutamol, an inhaled β2 agonist, is associated with a shorter duration and lower severity of ALI. A dose greater than 2.2 mg/day of inhaled salbutamol could be a minimal effective dose to evaluate in a randomized controlled trial.

## Introduction

Acute lung injury (ALI) is defined by impaired oxygenation (arterial partial pressure of oxygen/fraction of inspired oxygen (PaO_2_/FiO_2_) <300 mmHg) and bilateral infiltrates on a chest radiograph without clinical evidence of left atrial hypertension [1]. Pulmonary edema in ALI is caused by damage to the alveolar-capillary interface and increased permeability that leads to accumulation of protein rich edema fluid in the interstitial and alveolar spaces. Reabsorptive mechanisms to clear alveolar edema fluid are impaired in acute lung injury [2-4]. Furthermore, there is a dose effect indicated by the association of the greater degree of impaired edema clearance with longer duration of mechanical ventilation and decreased survival [5,6].

β2 agonists have several properties that could be beneficial in ALI. First, inhaled β2 agonists improve respiratory mechanics in patients with ALI as shown by decreased airflow resistance and peak airway pressures and increased dynamic compliance [7-9]. Second, β2 agonists have anti-inflammatory properties. β2-agonists attenuate the release of tumor necrosis factor-α and increase the production of IL-10 in response to endotoxin in animal models [10,11].

Intravenous dobutamine (which has β1 and β2 agonist action) attenuates pro-inflammatory cytokine expression in the lungs of a rat model of septic acute lung injury [12]. Third, β agonists increase alveolar edema fluid clearance in animal models of ALI [13-22], in the *ex vivo *human lung [19] and in patients with ALI [23]. Studies on the selective β blockers show that it is the β2 agonist activities that cause the enhanced edema fluid clearance [24].

To date, there have been no studies on the dose association of inhaled β agonists with duration or severity of human ALI. Our hypothesis was that a higher dose of inhaled β2 agonist use, compared to a lower dose, is associated with more days alive and free of ALI (a measure of duration of severity of ALI) in critically ill patients with acute lung injury.

## Materials and methods

This study was approved by the Research Ethics Board of Providence Health Care and the University of British Columbia, which waived the requirement of informed consent because of the retrospective nature of this study.

### Cohort of patients who had acute lung injury

Between September 2001 and August 2003, consecutive patients admitted to a tertiary care medical-surgical intensive care unit (ICU) at St Paul's Hospital, Vancouver, Canada, were screened and 86 of these met the American-European consensus conference definition of ALI who were on mechanical ventilation [1].

### Quantification of inhaled β2 agonist

Salbutamol was the only inhaled β2 agonist used clinically in the ICU. Salbutamol was administered through the ventilator circuit by metered dose inhaler (8 to 10 puffs at 100 μg/puff) or by nebulization of 2.5 to 5 mg of salbutamol solution (2.5 to 5 ml). The total daily dose of salbutamol administered and the route of delivery (metered dose inhaler or nebulizer) was recorded for each patient by retrospective chart review. We recorded salbutamol dose for each day in the ICU for 28 days or until discharge from the ICU (if less than 28 days). We calculated the average daily dose of salbutamol (mg/day) while in the ICU as the sum of total metered dose inhaler and nebulization dose (in mg) divided by the number of days in the ICU.

Several different doses of inhaled β2 agonists have been reported in mechanically ventilated patients [7,25,26]. Atabai and colleagues [27] measured levels of albuterol in plasma and broncho-alveolar lavage fluid from patients with ALI and found that doses of 2.5 mg or more of nebulized albuterol resulted in physiologically efficacious levels. In the only dose-response study published for mechanically ventilated patients, Dhand and colleagues [28] reported that a dose of 0.36 mg was as effective as 1.08 mg and 2.52 mg. This dose given every 4 hours would result in a total daily dose of 2.2 mg. Based on this, we classified patients receiving equal to or greater that 2.2 mg/day as 'high dose' and those patients receiving less than 2.2 mg/day as 'low dose'.

### Primary and secondary outcomes

The primary outcome was days alive and free of ALI over 28 days. Secondary outcomes were days alive and free of PaO_2_/FiO_2 _<300, days alive and free of cardiovascular, renal, hepatic, neurological, and hematological dysfunction, and 28-day mortality.

Organ dysfunction for each organ system was defined as being present during each 24 hour period if there was evidence of moderate, severe, or extreme organ dysfunction according to the Brussels criteria [29]. To assess duration of organ dysfunction and to correct organ dysfunction scoring for deaths in the 28-day observation period, we calculated days alive and free of organ dysfunction (DAF) as previously reported. Briefly, during each 24 hour period for each variable, DAF was scored as 1 if the patient was alive and free of organ dysfunction (normal or mild dysfunction). DAF was scored as 0 if the patient had organ dysfunction (moderate, severe, or extreme) or was not alive. Each of the 28 days after meeting the inclusion criteria was scored. A low score is indicative of more organ dysfunction because a low score indicates fewer days alive and free of organ dysfunction. Because data were not always available during the 24 hour period for each organ dysfunction variable, we used the carry forward assumption as defined previously [29]. For any 24 hour period in which there was no measurement of a variable, we carried forward the present or absent criteria from the previous 24 hour period. If any variable was never measured, it was assumed to be normal throughout the 28-day period.

Baseline demographics were age, gender, surgical versus medical diagnosis on admission to the ICU (based on the Acute Physiology and Chronic Health Evaluation (APACHE) III [30] diagnostic codes), admission APACHE II score [31], baseline PaO_2_/FiO_2 _ratio, history of chronic obstructive pulmonary disease (COPD), asthma, and/or smoking, cause of ALI (pulmonary versus extra-pulmonary), and proportion of patients that had sepsis or septic shock as defined by the ACCP/SCCM consensus conference [32].

### Statistical analysis

A comparison between the high and low dose salbutamol groups was made using the *t* test for continuous baseline demographic variables and outcomes. A chi-squared test was used for categorical variables. A forward selection multivariate linear regression model was constructed to evaluate the independence of salbutamol (high or low dose) against days alive and free of ALI. In the forward selection model, the following covariates were included: salbutamol (high or low dose), age (as a continuous variable), gender (female versus male), surgical versus medical diagnosis, history of COPD, asthma, and/or smoking, APACHE II score on admission (as a continuous variable), cause of ALI (pulmonary versus extrapulmonary), presence or absence of septic shock, and severity of ALI as defined by presence or absence of PaO_2_/FiO_2 _ratio ≤ 200. Variables were entered sequentially from the smallest to largest univariate *p* values and removed if they no longer met the inclusion cut-off after adjustment for the other variables. A two-tailed *p* value of <0.05 was used for statistical significance. The data were analyzed using SPSS 11.5 for Windows (SPSS Inc., Chicago, IL, USA, 2003). Continuous variables are presented as mean ± standard deviation unless otherwise stated.

## Results

The daily dose of salbutamol ranged from 0 to 6.4 mg/day. The cohort was divided into two groups using the cut-off point of 2.2 mg/day to compare the primary and secondary outcomes in those who received high dose salbutamol to those who received low dose. The mean salbutamol doses in the high and low dose groups were 3.72 mg/day and 0.64 mg/day respectively.

**Figure 1:**
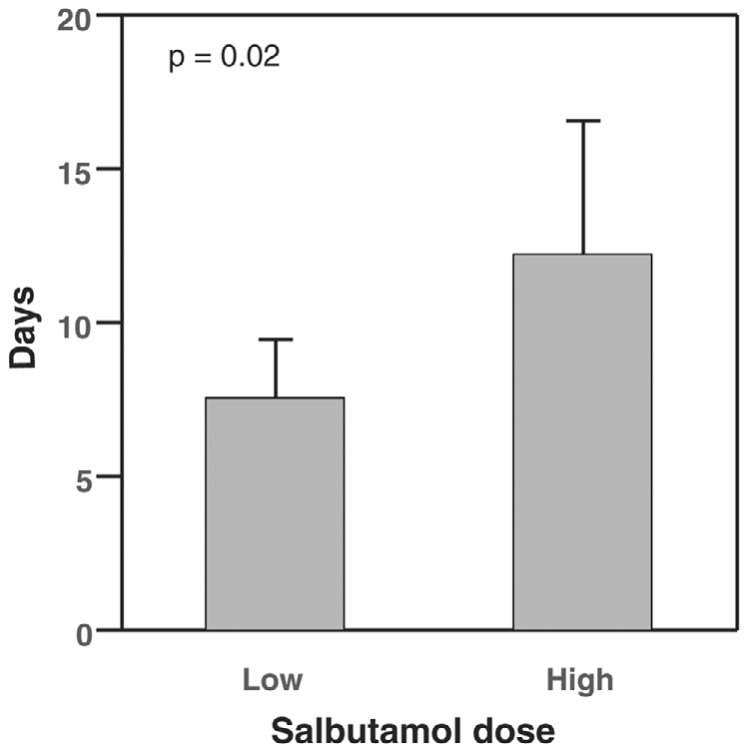
Days alive and free of acute lung injury in low dose (<2.2 mg/day) and high dose (≥ 2.2 mg/day) salbutamol groups (mean and 95% confidence interval).

**Figure 2:**
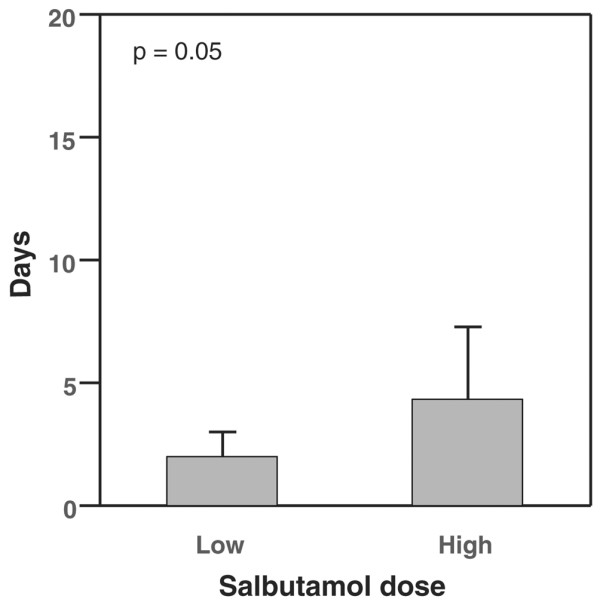
Days alive and free of PaO_2_/FiO_2 _<300 in low dose (<2.2 mg/day) and high dose (≥ 2.2 mg/day) salbutamol groups (mean and 95% confidence interval).

Patients who received high dose salbutamol had significantly more days alive and free of ALI (12.2 ± 4.4 days versus 7.6 ± 1.9 days, *p *= 0.02; Figure 1). Similarly, there was an association between the higher average daily dose of salbutamol and more days alive and free of PaO_2_/FiO_2 _ratio <300 (*p *= 0.05; Figure 2). There was no association between salbutamol dose and days alive and free of any of the non-pulmonary organ dysfunctions (Table 1). Mortality was not significantly different between the low and high dose groups (46.9% versus 50.0%, respectively).

The baseline demographics (Table 2) were similar between the groups except for a lower age in the low dose versus the high dose group (54.7 ± 16.6 years versus 65.7 ± 15.1 years, *p*< 0.05) and a lower proportion of patients with a history of COPD, asthma, and/or smoking in the low dose group versus the high dose group (15.6% versus 45.5%, *p*< 0.05).

Because of these differences at baseline between the two groups in age and in COPD/asthma/smoking status, a multivariate linear regression model was used to determine whether high dose salbutamol was independently associated with days alive and free of ALI when adjusting for other factors. High dose salbutamol remained a predictor of days alive and free of ALI in this model (*p *= 0.04). APACHE II score (*p *= 0.02) and cause of ALI (*p *= 0.02) were also independently associated with days alive and free of ALI (Table 3).

## Discussion

We found that high dose salbutamol, an inhaled β2 agonist, was associated with more days alive and free of ALI in critically ill patients who had ALI. This finding was supported by a similar significant association between dose of salbutamol and days alive and free of PaO_2_/FiO_2 _<300, a marker of severity of lung injury. Even after adjusting for differences in baseline characteristics between the high dose and low dose groups using a multivariate analysis, salbutamol was an independent predictor of more days alive and free of ALI.

Supporting the theory that β agonists have a direct effect on the pathophysiology of ALI, salbutamol dose was not significantly associated with days alive and free of any non-pulmonary organ dysfunction. To the best of our knowledge, this is the first study to show an association of the dose of an inhaled β-adrenergic agonist with a measure of duration of severity of ALI. Furthermore, this study suggests that a dose greater than 2.2 mg/day would be a reasonable dose to evaluate in a future prospective randomized controlled trial.

Our findings could be explained by one or more potentially beneficial actions of β2 agonists. β2 agonists such as salbutamol can improve pulmonary dysfunction in ALI by at least three mechanisms: increased alveolar fluid clearance, anti-inflammatory effects, and bronchodilation. The actions of β2 agonists in acute lung injury have recently been reviewed [33,34].

Stimulation of alveolar epithelial β2 receptors activates amiloride-sensitive sodium channels and ouabain-sensitive Na^+^/K^+^-ATPase to increase transepithelial sodium transport and alveolar fluid clearance via cAMP second messenger systems [35-37], which increases alveolar fluid clearance and alveolar epithelial function [38]. Beta-adrenergic agonists increase alveolar fluid clearance in normal lung [13-19] and in several animal models of acute lung injury [20-22] as well as in *ex vivo *human lungs [19] and in patients with ALI [23]. Terbutaline increases sodium transport across intact alveolar epithelium in isolated perfused rat lung, an effect that was inhibited by propranolol, indicating the importance of β receptor agonist activity [13]. Terbutaline also increases alveolar fluid clearance in anesthetized ventilated sheep [14], in dog lung [15], and in several models of ALI, such as hyperoxic lung injury [20], high tidal volume-associated lung injury [21] and the *in vivo *hypoxic rat model [22]. Resolution of alveolar edema is accelerated by isoproterenol [16,17,21] and epinephrine. Salmeterol, a specific β2 agonist, increased fluid clearance in both *ex vivo *human and rat lung [19]. In a recent double-blinded placebo controlled trial, intravenous salbutamol was shown to reduce extra vascular lung water in patients with ALI [23]. We did not measure lung water in our study so we cannot comment on whether salbutamol changed edema clearance in our study.

Beta-adrenergic agonists also have anti-inflammatory properties as β agonists decrease polymorphonuclear cell chemotaxis and accumulation in the lung [39] and decrease IL-1 [40], tumor necrosis factor-α [41] and IL-6 [42] production from macrophages. In a murine model of endotoxin-induced lung injury, dobutamine and dopexamine (both β1 and β2 agonists) decreased lung IL-6 protein and mRNA expression, and attenuated neutrophil accumulation in the lung [12]. We did not measure markers of inflammation in our study.

The third potential benefit of salbutamol on lung function in ALI is bronchodilation. β2 agonists decrease the elevated respiratory system resistance and airway pressure of patients who have acute respiratory distress syndrome (ARDS) [7-9]. In particular, both nebulized salbutamol (1 mg through the endotracheal tube) [7] and continuous intravenous infusion of salbutamol (15 μg/minute for at least 30 minutes) [9] decrease respiratory system resistance and airway pressure in ARDS. Wright and colleagues [8] also showed that a β2 agonist, aerosolized metaproterenol (5 mg), not only decreases high airway resistance and improves oxygenation, but also increases static compliance in human ARDS. This improvement of static compliance may be related to decreased lung edema or reduction in intrinsic positive end-expiratory pressure [7]. Overall, there may be clinical benefit from a reduction in respiratory resistance by β2 agonists in ALI because of a potential to decrease the risk of barotrauma.

There are few studies on the effects of β2 agonists on respiratory function in human ALI. Ware and Matthay [6] demonstrated that alveolar fluid clearance is impaired in most patients with ALI/ARDS and that impaired clearance is associated with a poor outcome. Basran and colleagues [43] studied the effect of intravenous terbutaline on plasma protein extravasation in ten patients with ALI/ARDS. Systemic terbutaline significantly reduced plasma transferrin movement into the lungs, a marker of lung permeability, in survivors but not non-survivors of ALI/ARDS. Perkins and colleagues [23] have recently reported that patients with ALI randomized to receive intravenous salbutamol (15 μg/kg/hr) for 7 days had a significant reduction in extra-vascular lung water index at days 4 and 7 compared to patients receiving placebo. They did not report any outcome data.

Several limitations of our study should be considered. First, there are limitations of retrospective studies such as ours. For example, the indications for salbutamol and the dose given were not controlled because our study was retrospective. Indeed, previous studies suggest even the high dose we defined (average of 3.7 mg/day) may be inadequate to attempt to increase alveolar fluid clearance. An alveolar concentration of 10^-6 ^M of salmeterol was associated with increased alveolar fluid clearance in an *ex vivo *human lung study [19]. An average dose of 3.5 ± 2.6 mg of albuterol in the previous 6 hours was associated with alveolar edema albuterol levels of 10^-6 ^M in patients who had ALI [27]. The intravenous dose Perkins and colleagues [23] reported is approximately ten-fold greater than our inhaled high dose threshold. A second limitation of our study is that other medications that can affect alveolar fluid clearance (such as infused catecholamines, diuretics, and corticosteroids) were not measured. However, Ware and Matthay [6] did not find a significant association between these medications and rate of edema fluid clearance. Therefore, these three medications may not have had a significant influence on alveolar fluid clearance in our patients. A third limitation is that our study was an association study that did not address mechanisms of improvement.

Finally, there were differences between the two dose groups in age and history of COPD, asthma and/or smoking, which could confound the association we found between the high dose of salbutamol and more days alive and free of ALI. To address this limitation, we did a multivariate analysis to adjust for differences in baseline characteristics. Importantly, the higher salbutamol dose remained independently associated with significantly more days alive and free of ALI even after multivariate analysis adjustment of baseline characteristics.

## Conclusion

This preliminary retrospective study demonstrates for the first time that the aerosolized β2 agonist salbutamol at a dose greater than 2.2 mg/day (average dose of 3.72 mg/day) given to mechanically ventilated patients with ALI was associated with more days alive and free of ALI. This possible beneficial association requires prospective studies, such as a rigorous randomized controlled trial, to determine whether inhaled β2 agonists improve relevant outcomes of ALI.

## Key messages

• 	β2 agonists have several properties that could be beneficial in ALI, including improving respiratory mechanics, reducing inflammation and increasing edema clearance.

• 	To date there have been no published studies examining the effect of β2 agonists on outcome from ALI.

• 	This retrospective study demonstrates an improved outcome from ALI with higher doses (average 3.72 mg/day) of inhaled salbutamol.

• 	A prospective randomized controlled trial examining the effect of β2 agonists on outcome from acute lung injury is required.

## Abbreviations

ALI = acute lung injury; APACHE = Acute Physiology and Chronic Health Evaluation; ARDS = acute respiratory distress syndrome; COPD = chronic obstructive pulmonary disease; DAF = days alive and free (of organ failure); FiO_2 _= fraction of inspired oxygen; ICU = intensive care unit; IL = interleukin; PaO_2 _= arterial oxygen partial pressure.

## Competing interests

The authors declare that they have no competing interests.

## Authors' contributions

SM, ES and HG collected and analyzed the data. ACG analyzed the data. KRW and JAR conceived and coordinated the study. All the authors contributed to, read and approved the final manuscript.

## Figures and Tables

**Table 1:** Comparison between the low versus high dose salbutamol groups and non-pulmonary organ dysfunction and mortality

Days alive and free	Low dose (n = 64)^a^	High dose (n = 22)^a^	*p* value
Cardiovascular	11.5 ± 2.6	13.2 ± 4.2	0.50
Renal	14.3 ± 2.9	16.0 ± 4.5	0.55
Hepatic	17.4 ± 2.8	19.6 ± 4.4	0.42
Hematological	15.9 ± 2.9	19.6 ± 4.5	0.10
Neurological	16.6 ± 2.6	19.0 ± 3.9	0.35
Mortality (%)	46.9%	50.0%	0.80

^a^Values are mean ± standard error of the mean.

**Table 2:** Baseline characteristics of patients who had acute lung injury

Characteristic	Low dose (n = 64)	High dose (n = 22)	*p* value
Mean salbutamol dose (mg/day, range)	0.64 (0–2.19)	3.72 (2.2–6.4)	<0.001
Age (years)	54.7 ± 16.6	65.7 ± 15.1	0.007
Gender (% female)	41%	45%	0.70
Surgical diagnosis	31.3%	31.8%	0.96
APACHE II	27.2 ± 8.1	25.2 ± 7.1	0.27
Cause of ALI			
Pulmonary	51.6%	50%	
Extra-pulmonary	48.4%	50%	0.90
History of COPD, asthma, and/or smoking	15.6%	45.5%	0.007
PaO_2_/FiO_2 _≤ 200	81.3%	68.2%	0.20
Sepsis	95%	100%	0.41
Septic shock	81.3%	72.7%	0.29

ALI, acute lung injury; COPD, chronic obstructive pulmonary disease; FiO_2_, fraction of inspired oxygen; PaO_2_, arterial oxygen partial pressure.

**Table 3:** Multivariate linear regression model for prediction of days alive and free of acute lung injury

Covariates	β (slope)	95% CI of β		*p* value
Salbutamol (high dose)	4.08	0.17	8.00	0.04
APACHE II	-0.25	-0.47	-0.03	0.02
Cause of ALI	-3.96	-7.37	-0.56	0.02

Covariates: salbutamol (high referenced to low dose), age (as a continuous variable), gender (female referenced to male), surgical versus medical diagnosis, history of chronic obstructive pulmonary disease, asthma, and/or smoking, APACHE II score on admission (as a continuous variable), cause of acute lung injury (ALI; pulmonary referenced to extrapulmonary), presence or absence of septic shock, and severity of ALI as defined by presence or absence of arterial oxygen partial pressure/fraction of inspired oxygen (PaO_2_/FiO_2_) ratio ≤ 200. CI, confidence interval.
